# Participants with Normal Weight or with Obesity Show Different Relationships of 6-n-Propylthiouracil (PROP) Taster Status with BMI and Plasma Endocannabinoids

**DOI:** 10.1038/s41598-017-01562-1

**Published:** 2017-05-02

**Authors:** Gianfranca Carta, Melania Melis, Stefano Pintus, Paolo Pintus, Carla A. Piras, Laura Muredda, Daniela Demurtas, Vincenzo Di Marzo, Sebastiano Banni, Iole Tomassini Barbarossa

**Affiliations:** 1 Department of Biomedical Sciences, Section of Physiology, University of Cagliari, Monserrato, CA Italy; 2 Center for Metabolic Diseases, Internal Medicine Department–A.O., Brotzu, Cagliari Italy; 3 Endocannabinoid Research Group, Institute of Biomolecular Chemistry, Consiglio Nazionale delle Ricerche, Pozzuoli, NA Italy

## Abstract

Reduced taste sensitivity to 6-n-propylthiouracil (PROP), a genetic trait regarded as a general index for oral chemosensory perception, has been associated with a calorie-rich food preference and lower circulating endocannabinoid levels in participants with normal weight (NW), which suggests an adaptive mechanism to maintain a lean phenotype. In this study, we assessed whether participants with obesity (OB) show different patterns of plasma endocannabinoids and lipid metabolism biomarkers from those of NW, with further categorization based on their PROP sensitivity. NW and OB were classified by their PROP taster status as non-tasters (NT), medium-tasters (MT) and supertasters (ST). The blood samples were analysed for plasma endocannabinoids, nonesterified fatty acids (NEFA) and retinol, which have been associated to metabolic syndrome. In OB, we found a higher BMI and lower circulating endocannabinoids in ST vs. OB NT. However, OB ST showed lower circulating NEFA and retinol levels, which suggested a more favourable lipid metabolism and body fat distribution than those of OB NT. We confirmed lower plasma endocannabinoid levels in NW NT than in NW ST. These data suggest that PROP taste sensitivity determines metabolic changes and ultimately body mass composition differently in OB and NW.

## Introduction

Optimal body fat deposition is crucial to maintain the most favourable body composition to fulfil physiological needs, and it depends on the balance between energy expenditure and food intake. Inadequate food choices may modify and override physiological signals that regulate dietary intake. Taste sensitivity is one of the most important determinants influencing food preferences and therefore dietary behaviour and metabolism^1–3^. Peculiarly, the genetic trait of taste sensitivity to the bitter compound 6-n-propylthiouracil (PROP) has been proposed as a marker for individual differences in taste perception, general food preferences and dietary behaviour with subsequent links to body mass composition^1, 2, 4–7^. PROP non-taster (NT) individuals, who show little or no taste responsiveness to PROP, seem to have a higher preference to consume high-fat/high-energy and/or strong-tasting foods^4–6, 8, 9^. In contrast, participants who are moderately or very sensitive to PROP (medium tasters (MT) and supertasters (ST), respectively) show greater sensitivity and a lower liking for and intake of these foods^1, 10, 11^. These associations support the hypothesis that PROP tasting is inversely related to food intake, calorie consumption and body weight, which have been reported in several studies^1, 9, 12–15^. However, other studies did not confirm these associations^16–20^. Therefore, it is likely that other genetic and non-genetic factors may contribute to the increased predisposition to higher energy intake in NT participants^3^. Recently, we proposed that the lower endocannabinoid levels observed in normal weight (NW) NT than in ST represent an adaptive mechanism that is an attempt to normalize various feeding behaviours in these participants, such as the increased disinhibition typical of NT, and thus explains, in part, why they still exhibit a lean phenotype despite their putative preference for calorie-dense foods^21^. However, it is not known whether this potential physiological mechanism is disrupted in the conditions of excess body fat or whether food choice, when determined more by taste sensitivity than physiological needs, may lead to a long-term positive energy balance and thereby to obesity in a different manner in ST than in NT. Indeed, it is known that cognitive control of eating behaviour can play a prominent role in determining the relationship between PROP taster status and BMI^3^.

Obesity is characterized by an overactive endocannabinoid system with increased plasma levels of the endocannabinoids anandamide (AEA) and 2-arachidonoylglycerol (2-AG)^22^. Pharmacological strategies to block the endocannabinoid system using CB1 receptor antagonists have been successful at reducing body fat mass and improving obesity-associated metabolic disorders^23^. The changes in the plasma endocannabinoid level may also be influenced by the quality of the dietary fat^24^. However, in our previous study on NW, changes in endocannabinoid levels associated with differences in PROP sensitivity did not correspond to large differences in short-term qualitative fatty acid intake^21^. However, in participants with obesity (OB), a chronically disrupted lipid metabolism and/or an unbalanced dietary regimen may influence endocannabinoid biosynthesis^24^.

In this study, we aimed to evaluate whether NW and OB, based on their PROP sensitivity, showed different patterns of plasma endocannabinoids and congeners such as palmitoylethanolamine (PEA), implicated in obesity-associated low grade inflammation^25^, and lipid metabolism biomarkers, such as (1) circulating free fatty acids (FFA) and retinol, both of which have been shown to increase in subjects with visceral obesity and the related insulin resistance^26, 27^; (2) the erythrocyte fatty acid profile, which may reflect either the qualitative dietary fatty acid intake^28^ and excessive carbohydrate intake through *de novo lipogenesis* (DNL) with increased saturated fatty acids (SAFA) in tissues^29^ and its specific biomarker linoleic (LA) palmitic acid (PA) ratio^30^; (3) plasma beta-carotene as a marker of dietary intake of fruits and vegetables^31, 32^, in order to evaluate whether changes in the plasma endocannabinoid level may also be influenced by the quality of the dietary fat and/or an unbalanced dietary regimen. In addition, we also evaluated whether BMI changes in relation to PROP status, was associated with other two parameters known to influence both lipid metabolism and endocannabinoid levels, waist/hip ratio and Type 2 diabetes incidence. The assessment of the general eating attitudes of NW and OB according to their PROP taster status was as well evaluated since our previous results showed that the disinhibition score of NT was higher than those ST in NW participants^21^.

## Results

### Participants’ anthropometric and eating behaviour parameters in association with PROP taster status

The values (mean ± SD) of anthropometric parameters of participants with normal weight and obesity are shown in Table 1.

**Table 1:** Anthropometric parameters of participants with normal weight (NW) or with obesity (OB).

	NW *N* = 60	OB *N* = 50	*P*
Age (y)	46.27 ± 13.44	51.36 ± 15.46	0.07
BMI (kg/m^2^)	22.02 ± 2.10	35.97 ± 5.11	0.00
Waist (cm)	75.77 ± 8.87	106.78 ± 11.94	0.00
Hip (cm)	94.83 ± 5.71	120.17 ± 11.61	0.00
W/H ratio	0.80 ± 0.07	0.89 ± 0.08	0.00
Leptin (ng/ml)	5.04 ± 3.88	20.98 ± 12.09	0.00

Values (mean ± SD); *P* was assessed using an unpaired, two-tailed Student’s t-test.

**Figure 1:**
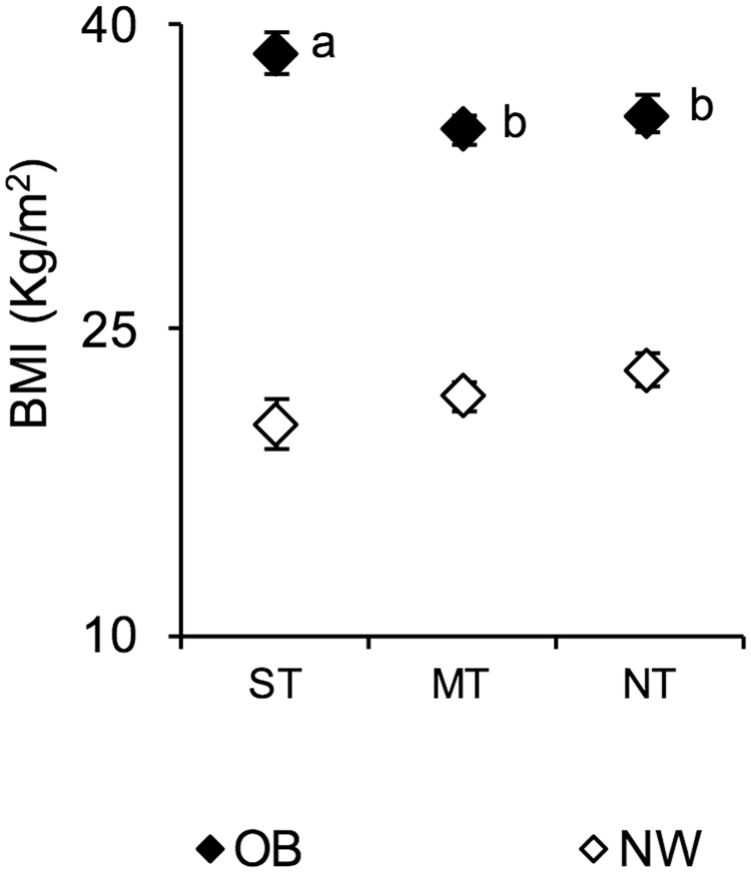
Mean values ± SEM of body mass index (BMI) of supertaster (*N* = 9), medium taster (*N* = 28) and non-taster (*N* = 23) participants with normal weight (NW) and supertaster (*N* = 12), medium taster (*N* = 25) and non-taster (*N* = 13) participants with obesity (OB). A two-way analysis of variance (ANOVA) was used to compare BMIs across taster groups in participants with normal weight or with obesity (*P* = 0.013). The different letters indicate significant differences (*P* < 0.023; Newman-Keuls test). ST, supertaster participants; MT, medium-taster participants; and NT, non-taster participants.

The mean values ± SEM of BMI in NW and OB according to PROP taster status are shown in Fig. 1. An ANOVA revealed a significant two-way interaction of the Taster group × NW/OB status for the BMI values (*F*_[2,104]_ = 4.556; *P* = 0.013). Post hoc comparisons showed that the BMI of ST OB was significantly higher than that of MT and NT OB (*P* = 0.013 and *P* = 0.023; Newman-Keuls test). We also considered whether the waist/hip ratio changed according to PROP status within NW or OB, and we did not find any significant difference. In addition, we also investigated whether there was a higher incidence of type 2 diabetes in the OB NT cohort. We found that although the difference was not statistically significant (chi squared = 1.5; *P* = 0.47), 25% of ST, 44% of MT and 46% of NT of OB subjects were affected by type 2 diabetes.

The scores of the 3-Factor Eating Questionnaire by Stunkard & Messick^33^ (TFEQ) for the assessment of the general eating attitudes of NW and OB according to their PROP taster status are shown in Table 2. The scores for disinhibition were higher for OB than NW for all three taster groups (*P* < 0.049; Newman-Keuls test subsequent two-way ANOVA), whereas no significant differences in the disinhibition scores were found according to the PROP taster status. No changes were found in the restraint or hunger scores between OB and NW or among the various PROP tasters (data not shown).

**Table 2:** Scores for dietary restraint, disinhibition, and perceived hunger of supertasters (ST), medium tasters (MT) and non-tasters (NT) for both participants with normal weight (NW) or with obesity (OB).

	PROP sensitivity		
	ST	MT	NT
**NW**			
Restraint	11.11 ± 1.15	9.46 ± 0.85	11.22 ± 0.94
Disinhibition	3.33 ± 1.00*	3.35 ± 0.56*	4.91 ± 0.63*
Hunger	5.37 ± 1.06	2.94 ± 0.70	3.47 ± 0.77
**OB**			
Restraint	12.66 ± 1.31	13.44 ± 0.91	10.38 ± 1.25
Disinhibition	8.17 ± 0.87	7.52 ± 0.60	7.07 ± 0.83
Hunger	5.27 ± 0.90	6.38 ± 0.70	4.5 ± 1.22

The scores (mean ± SEMs) are determined by the Three-Factor Eating Questionnaire^33^. *N* = 110. *A significant difference between participants with normal weight or with obesity (*P* < 0.049; Newman-Keuls test subsequent to the two-way ANOVA).

### Erythrocyte fatty acid profile

Table 3 shows the erythrocyte fatty acid profile expressed as mol%, in NW and OB. We found a 13% increase in SAFA with a major contribution of PA at the expense of polyunsaturated fatty acids (PUFA) and mainly linoleic acid LA in OB compared with NW. In fact, the ratio of PA/LA was 28% higher in OB than in NW. This ratio has been used as a biomarker of DNL^30^. The only SAFA measured that did not differ between NW and OB was pentadecanoic acid, which was previously suggested to be a biomarker of dietary dairy products^34^. To confirm these data, we also measured other typical biomarkers of dairy product intake, such as c9, t11 conjugated linoleic acid and vaccenic acid^35^, and typical biomarkers of omega-3 intake such as EPA and DHA levels^36^, but we did not find any significant differences between OB and NW (data not shown).

**Table 3:** Erythrocyte fatty acid profile and plasma beta-carotene level of participants with normal weight (NW) or with obesity (OB).

	NW *N* = 60	OB*N* = 50	*P*
12:0	0.18 ± 0.10	0.24 ± 0.15	0.01
14:0	0.68 ± 0.36	0.94 ± 0.40	0.00
15:0	0.68 ± 0.28	0.82 ± 0.38	0.07
16:0	28.17 ± 7.46	32.13 ± 2.25	0.00
18:0	10.82 ± 2.94	12.24 ± 1.45	0.02
16:1n-9	2.05 ± 0.48	2.01 ± 0.39	0.69
18:1n-9	17.53 ± 3.69	16.93 ± 2.43	0.35
18:2n-6	16.12 ± 4.04	12.57 ± 2.31	0.00
18:3n6	0.18 ± 0.09	0.21 ± 0.09	0.18
20:3n-6	1.66 ± 0.49	1.55 ± 0.30	0.13
20:4n-6	13.68 ± 2.93	12.65 ± 1.72	0.05
22:4n-6	1.80 ± 0.60	1.61 ± 0.45	0.11
22:5n-6	0.26 ± 0.12	0.23 ± 0.08	0.18
18:3n3	0.17 ± 0.06	0.18 ± 0.12	0.41
20:5n-3	0.68 ± 0.31	0.76 ± 0.49	0.21
22:5n3	0.96 ± 0.31	0.84 ± 0.18	0.05
22:6n-3	4.08 ± 1.08	3.79 ± 0.88	0.29
SAFA	40.57 ± 10.45	46.46 ± 2.51	0.00
MUFA	19.59 ± 3.90	18.94 ± 2.42	0.34
PUFA	39.85 ± 7.32	34.60 ± 2.38	0.00
PA/LA	1.92 ± 0.76	2.65 ± 0.58	0.00
Omega-3 index	5.72 ± 1.48	5.39 ± 1.34	0.45
Beta-carotene	0.98 ± 0.66	0.54 ± 0.33	0.00

The erythrocyte fatty acids are expressed as mol% of total fatty acids, whereas the beta-carotene is expressed as nmoles/ml plasma. Mean ± SD; *P* was assessed using an unpaired, two-tailed Student’s t-test.

### Endocannabinoids and the palmitoylethanolamine (PEA) profile

**Figure 2:**
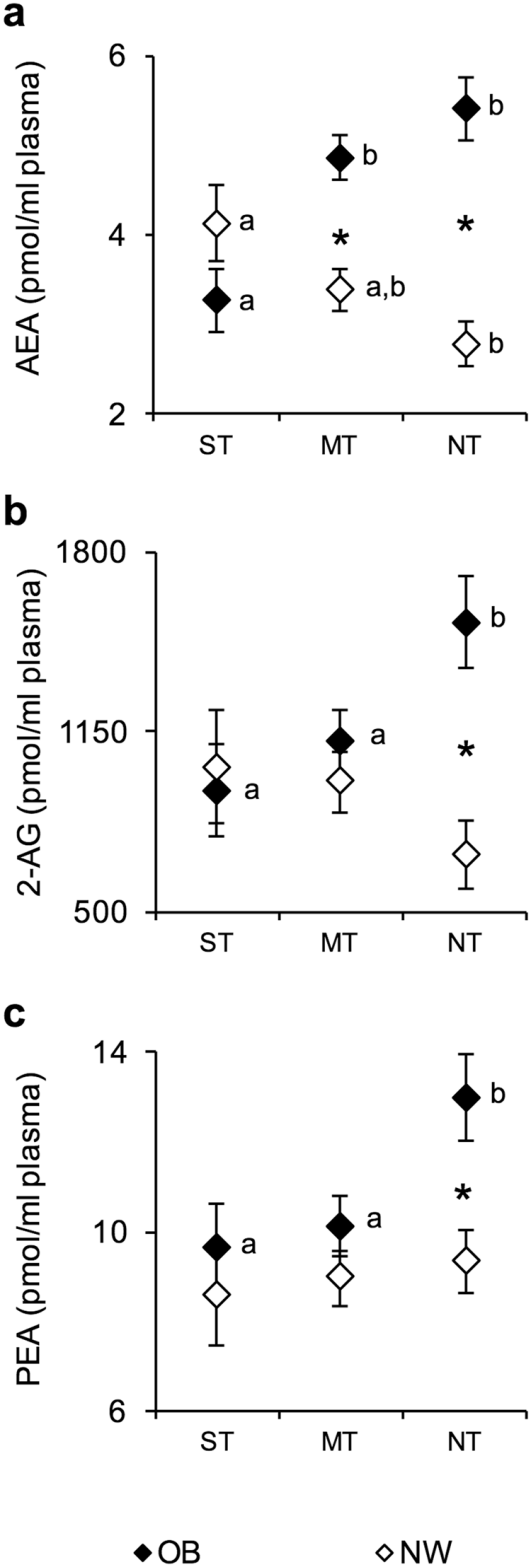
Mean values ± SEM of endocannabinoids (AEA and 2-AG) and the palmitoylethanolamide (PEA) congener plasma levels measured in supertaster (*N* = 9), medium taster (*N* = 28) and non-taster (*N* = 23) participants with normal weight (NW) and supertaster (*N* = 12), medium taster (*N* = 25) and non-taster (*N* = 13) participants with obesity (OB). (**a**) Anandamide (AEA) values. (**b**) 2-arachidonoylglycerol (2-AG) values. (**c**) PEA values. A two-way analysis of variance (ANOVA) was used to compare the differences across taster groups in participants with normal weight or with obesity (AEA and 2-AG: *P* < 0.009). The different letters indicate significant differences (AEA: *P* < 0.019; 2-AG: *P* = 0.044; and PEA: *P* = 0.020; Newman-Keuls test). *A significant difference between participants with normal weight or with obesity (*P* < 0.017; Newman-Keuls test). ST, supertaster participants; MT, medium taster participants; and NT, non-taster participants.

The plasma levels of the endocannabinoids AEA and 2-AG, and the AEA congener PEA (mean values ± SEM) were measured in NW and OB according to the PROP taster status and are shown in Fig. 2. An ANOVA revealed a significant two-way interaction of the Taster group × NW/OB status for the AEA or 2-AG values (*F*_[2,104]_ = 12.248; *P* < 0.0000 or *F*_[2,104]_ = 4.914; *P* = 0.009) (Fig. 2a and b). Post hoc comparisons showed that AEA plasma levels of ST OB were significantly lower than those of MT and NT OB (*P* = 0.003 and *P* = 0.0002; Newman-Keuls test), whereas those of ST NW were significantly higher than those of NT NW (*P* = 0.019; Newman-Keuls test) (Fig. 2a). The AEA plasma levels of MT and NT NW were significantly lower than those of the corresponding OB (*P* = 0.0043 and *P* = 0.00012; Newman-Keuls test) (Fig. 2a). The 2-AG plasma levels of ST and MT OB were significantly lower than those of NT OB (*P* = 0.044; Newman-Keuls test) who had levels higher than those of NT NW (*P* = 0.0024; Newman-Keuls test) (Fig. 2b). The associations between PROP taster group and AEA and 2AG on NW and OB remained after controlling by BMI, or after controlling for waist to hip ratio as tested by two-way ANCOVA analysis (data not shown). A pairwise comparison also showed that the PEA plasma levels of NT OB were significantly higher than those of MT and ST OB (*P* = 0.0207 and *P* = 0.0192; Newman-Keuls test) and of NT NW (*P* = 0.0179; Newman-Keuls test) (Fig. 2c). In addition, no changes were found in plasma oleoylethanolamide (OEA) levels (data not shown).

### Lipid parameters in erythrocytes and plasma related to PROP taster status

**Figure 3:**
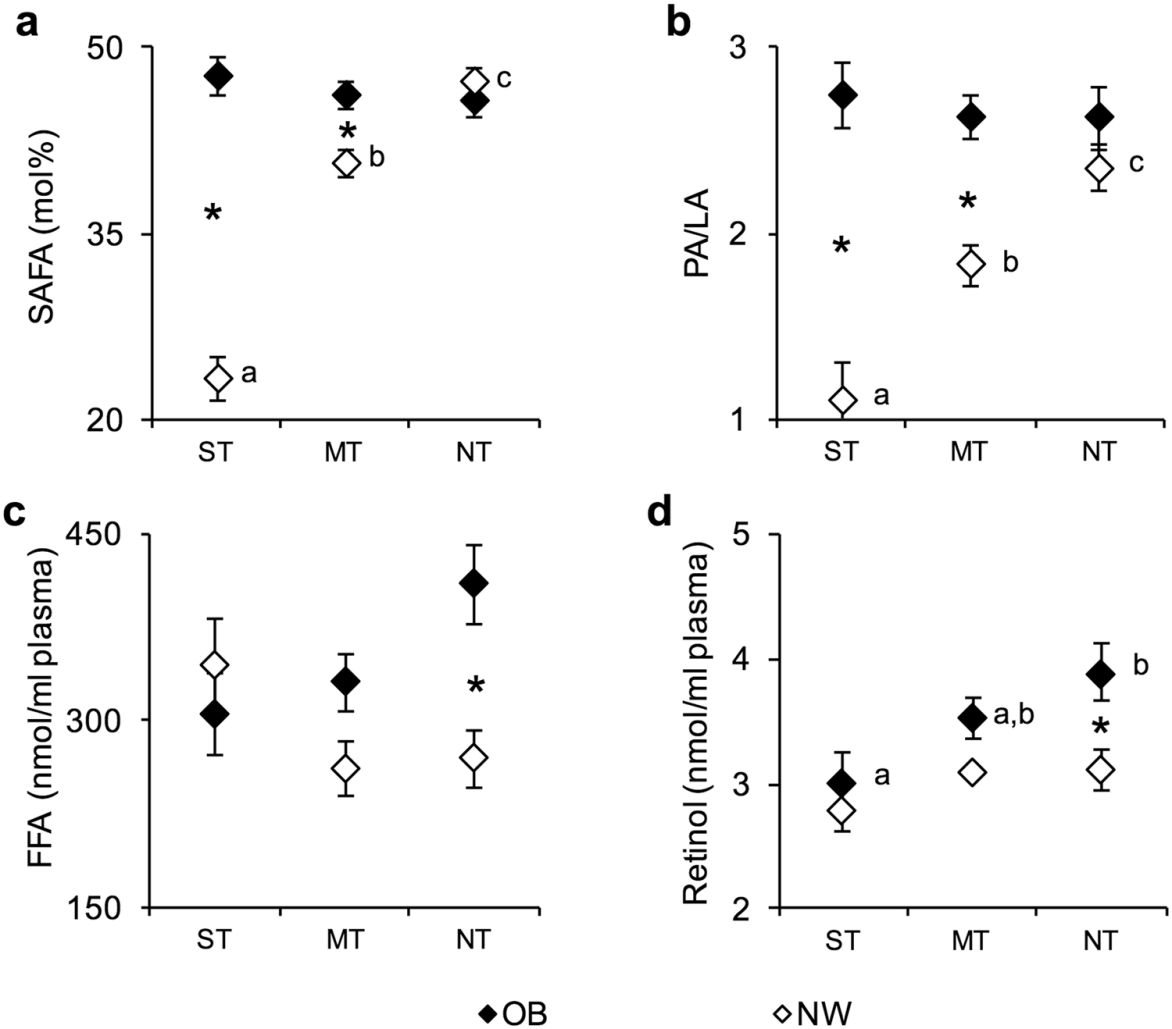
Mean values ± SEM of erythrocyte saturated fatty acid (SAFA) levels and the palmitic/linoleic acids ratio (PA/LA), plasma levels of free fatty acid (FFA) and retinol measured in supertaster (*N* = 9), medium taster (*N* = 28) and non-taster (*N* = 23) participants with normal weight (NW) and supertaster (*N* = 12), medium tasters (*N* = 25) and non-taster (*N* = 13) participants with obesity (OB). (**a**) Erythrocyte SAFA values. (**b**) Erythrocyte palmitic/linoleic acids ratio (PA/LA). (**c**) Plasma free fatty acid (FFA) values. (**d**) Plasma retinol values. A two-way analysis of variance (ANOVA) was used to compare the differences across taster groups in participants with normal weight or with obesity (SAFA, PA/LA: *P* < 0.00068 and FFA: *P* = 0.0243). The different letters indicate significant differences (SAFA: *P* < 0.0069; PA/LA: *P* < 0.0214; retinol: *P* = 0.0333; Newman-Keuls test). *A significant difference between participants with normal weight or with obesity (*P* < 0.0265; Newman-Keuls test). ST, supertaster participants; MT, medium taster participants; and NT, non-taster participants.

The levels of erythrocytes SAFA (mean values ± SEM), PA/LA ratio, plasma FFA and retinol measured in NW and OB according to the PROP taster status are shown in Fig. 3. A significant two-way interaction of Taster group × NW/OB status on SAFA values or PA/LA or FFA values was found (*F*_[2,104]_ = 36.901, *P* = 0.0000; *F*_[2,104]_ = 7.8328, *P* = 0.00068 and *F*_[2,104]_ = 3.8502, *P* = 0.0243; two-way ANOVA). Post hoc comparisons showed that the SAFA values (Fig. 3a) and the PA/LA ratio (Fig. 3b) of ST NW were significantly lower than those of MT NW (*P* = 0.00011 and *P* = 0.0015, respectively; Newman-Keuls test), which also had levels lower than those of NT NW (*P* = 0.0069 and *P* = 0.0214, respectively; Newman-Keuls test). The SAFA values and the PA/LA ratio of ST and MT NW were significantly lower than those of the corresponding OB (SAFA values*: P* = 0.00012 and *P* = 0.0161, respectively; PL/LA: *P* = 0.00012 and *P* = 0.0032, respectively; Newman-Keuls test). Post hoc comparisons also showed that the FFA plasma levels of NT OB were significantly higher than those of NT NW (*P* = 0.0082; Newman-Keuls test) (Fig. 3c). The plasma retinol level of ST OB was lower than those of NT OB (*P* = 0.0333; Newman-Keuls test), which had levels higher than those of NT NW (*P* = 0.0265; Newman-Keuls test) (Fig. 3d).

**Figure 4:**
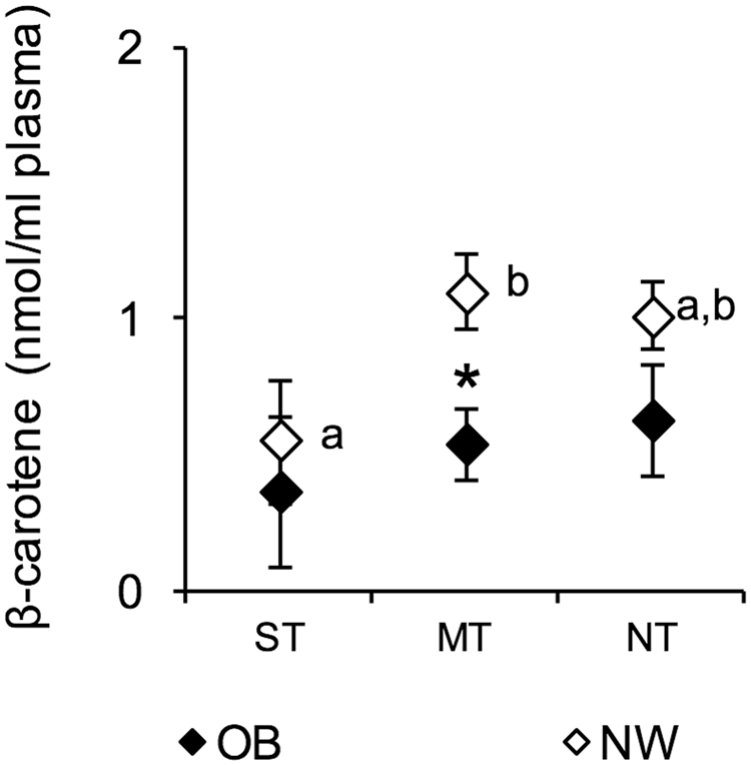
Mean values ± SEM of the plasma levels of beta-carotene measured in supertaster (*N* = 9), medium taster (*N* = 28) and non-taster (*N* = 23) participants with normal weight (NW) and supertaster (*N* = 12), medium taster (*N* = 25) and non-taster (*N* = 13) participants with obesity (OB). ST, supertaster participants; MT, medium taster participants; and NT, non-taster participants. The different letters indicate significant differences (*P* = 0.0409; Newman-Keuls test subsequent to the two-way ANOVA). *A significant difference between participants with normal weight or with obesity (*P* = 0.0044; Newman-Keuls test subsequent to the two-way ANOVA).

ST NW showed plasma levels of beta-carotene that were lower than those of MT NW (*P* = 0.0409; Newman-Keuls test subsequent to a two-way ANOVA), which had levels higher than those of MT OB (*P* = 0.0044; Newman-Keuls test subsequent to a two-way ANOVA) (Fig. 4).

The results of the data analysis are referred to both genders, however, when limited to women, did not change significantly (data not shown).

Table 4 shows the effect size and power calculated for each dependent variable after conducting two-way ANOVA with PROP taste and NW /OB status as independent variables.

**Table 4:** Effect size and Power calculated for each dependent variable (DV) after conducting two-way ANOVA with PROP taste and NW /OB status as independent variables.

DV		*SS*	*df*	*MS*	*F*	*p*	Eta^2^	Power
BMI	NW/OB status	4959.140	1	4959.140	376.618	0.000	0.765	1.000
	Taster group	33.320	2	16.660	1.265	0.286	0.005	0.283
	NW/OB status × Taster group	120.000	2	60.000	4.557	0.013	0.018	0.788
	Error	1369.430	104	13.170				
	Total	6481.89	109					
AEA	NW/OB status	25.370	1	25.370	17.052	0.000	0.117	0.989
	Taster group	2.741	2	1.371	0.921	0.401	0.013	0.219
	NW/OB status × Taster group	36.447	2	18.223	12.248	0.000	0.168	0.997
	Error	151.760	104	1.488				
	Total	216.318	109					
2AG	NW/OB status	1962770	1	1962770	5.845	0.017	0.049	0.701
	Taster group	276661	2	138331	0.412	0.663	0.007	0.121
	NW/OB status × Taster group	3300754	2	1650377	4.914	0.009	0.083	0.827
	Error	34254081	104	335824				
	Total	39794266	109					
PEA	NW/OB status	86.444	1	86.444	7.738	0.006	0.064	0.813
	Taster group	69.538	2	34.769	3.112	0.049	0.052	0.618
	NW/OB status × Taster group	34.864	2	17.432	1.561	0.215	0.026	0.344
	Error	1150.611	104	11.171				
	Total	1341.457	109					
SAFA	NW/OB status	2081	1	2081.000	70.825	0.000	0.235	1.000
	Taster group	1551	2	775.500	26.394	0.000	0.175	1.000
	NW/OB status × Taster group	2168.5	2	1084.200	36.901	0.000	0.245	1.000
	Error	3055.7	104	29.400				
	Total	8856.2	109					
PA/LA	NW/OB status	18.697	1	18.697	49.080	0.000	0.273	1.000
	Taster group	4.153	2	2.077	5.451	0.006	0.061	0.859
	NW/OB status × Taster group	5.968	2	2.984	7.833	0.000	0.087	0.958
	Error	39.619	104	0.381				
	Total	68.4369	109					
FFA	NW/OB status	76352	1	76352	5.840	0.017	0.048	0.692
	Taster group	40484	2	20242	1.548	0.218	0.025	0.339
	NW/OB status × Taster group	100667	2	50334	3.850	0.024	0.064	0.713
	Error	1359600	104	13073				
	Total	1577103	109					
retinol	NW/OB status	5.326	1	5.326	7.849	0.006	0.065	0.814
	Taster group	4.517	2	2.258	3.328	0.040	0.055	0.645
	NW/OB status × Taster group	1.098	2	0.549	0.809	0.448	0.013	0.194
	Error	70.577	104	0.679				
	Total	81.5183	109					
beta-carotene	NW/OB status	1.767	1	1.767	5.737	0.019	0.077	0.875
	Taster group	1.072	2	0.536	1.741	0.184	0.047	0.570
	NW/OB status × Taster group	0.294	2	0.147	0.478	0.622	0.012	0.187
	Error	19.713	104	0.308				
	Total	22.8462	109					

SS, sum of squares; df, degree of freedom; MS, mean square; Eta^2^, parameter showing the effect size.

The effect size was very large for PROP taste and NW /OB status and AEA and SAFA, medium in the case of 2AG, PA/LA and FFA and small in case of BMI, PEA, retinol and beta-carotene in according to Cohen’s guidelines (1988). However, the same analyses in the case of PEA, retinol and beta-carotene resulted underpowered, at the limit of significance in case of BMI and FFA, and had sufficient power in the case of AEA, 2AG, SAFA and PA/LA.

## Discussion

We investigated the different profiles of plasma endocannabinoid levels in NT, MT and ST according to BMI by comparing obese vs. lean participants. Previously^21^, and in view of the general positive effects of the endocannabinoid system on food intake and fat accumulation in mammals and humans^22^, we suggested that the lower concentrations of AEA and 2-AG in the plasma of NT vs. ST in NW participants might represent an adaptive mechanism to counteract excess fat accumulation. This mechanism may derive from a putative increased intake of high energy food in the former vs. the latter population and explain why NT can still be lean. In this study, we confirmed the previous data in a different cohort of NW participants and observed a trend toward higher BMI in NW NT than in ST, and we confirmed the propensity of the former to accumulate more body weight than the latter. Perhaps more importantly, we showed that in OB volunteers, the opposite correlation was found between circulating AEA and 2-AG levels and the PROP phenotype with NT having approximately 62% higher concentrations of both endocannabinoids than ST. This finding suggests that the obesity state disrupts the aforementioned adaptive mechanisms and possibly creates the conditions for an endocannabinoid-mediated “obesity vicious circle” in NT individuals. Accordingly, OB NT exhibited higher plasma levels of both AEA and 2-AG than NW NT, which suggests that in an obese population, the NT phenotype might also contribute to enhanced body fat via an overactive endocannabinoid system. In contrast, when passing from NT to MT and ST, the difference in circulating endocannabinoid levels between OB and NW gradually diminishes to become non-statistically significant in ST. This finding may indicate that the co-occurrence of the ST and OB phenotypes renders the role of the endocannabinoid system less important in contributing to obesity and conversely to the capability of a deficiency in this system to counteract obesity. Accordingly, the difference in BMI between OB and NW was higher in ST than in NT.

The higher BMI associated with lower endocannabinoid levels in OB ST might be related to a different adiposity distribution with a relatively lower proportion of visceral adipose tissue (VAT) than in OB NT. In fact, the plasma concentration of endocannabinoids has been positively associated with increased visceral adiposity in obese humans^37, 38^ and experimental animals^39, 40^. Indeed, a higher proportion of female participants, who usually exhibit a constitutively lower proportion of VAT than males, were present in our OB ST population than in the OB NT population. When we restricted the analysis exclusively to OB females, we still found higher BMI and lower endocannabinoids levels in ST than in NT, although these data should be confirmed in a larger population. Further support of the hypothesis of a different body fat distribution between ST and NT is that the FFA and circulating retinol concentrations were higher in OB NT than in ST. In fact, increased plasma levels of both FFA and retinol-binding protein (RBP) have been associated with a higher proportion of VAT^26, 27^. Additionally, the increase in PEA in NT OB may be due to an enhanced inflammatory tone^41^ induced by a higher proportion of VAT, which is more likely to result in macrophage infiltration than subcutaneous adipose tissue (SAT) during obesity. Furthermore, although not statistically significant, we detected a slight higher waist/hip ratio and higher type 2 diabetes incidence in OB NT relative to OB ST further suggests that OB ST, despite having higher BMI, tends to have a more favourable body fat distribution and thereby a lower type 2 diabetes incidence. Future studies will be devoted to establish, by CT scan or MRI, whether PROP taste sensitivity is associated with a different body fat distribution. Because the preferable SAT fat accumulation has been associated with metabolic healthy obesity (MHO)^42, 43^, future studies in a large cohort of participants (with an extended follow up) should also investigate whether OB ST are more protected from dysmetabolic conditions and more resistant to metabolic disorders.

Obesity is usually characterized by a modification of tissue lipid deposition and the fatty acid profile determined by metabolic changes and/or an imbalance of nutrient intake. The increases in the SAFA concentration and the PA/LA ratio in erythrocytes and the decrease in beta-carotene plasma concentrations found in the present study in OB with respect to NW may suggest an excessive intake of carbohydrates and a lower consumption of vegetables and fruits. In fact, increases in SAFA and PA/LA ratio, rather than an increase in dietary SAFA, indicate sustained DNL^30^, which has been associated with excess carbohydrate intake^29^. Notably, the detection of increased levels of these two parameters in erythrocytes suggests that these changes reflect a long-term modification of tissue phospholipid fatty acid profiles. Beta-carotene has been widely used as a marker of vegetable intake^31, 32^, and a recent dietary survey in Italy^44^ showed that vegetables and fruits contribute to plasma level concentrations of beta-carotene of 67.8% and 22.3%, respectively, and therefore account for approximately 90% of the beta-carotene concentration. If the changes detected in erythrocyte fatty acids and plasma beta-carotene may be considered biomarkers of nutritional and metabolic imbalance, our data confirm that the PROP phenotype is accompanied by long-term changes in nutrient intake. In fact, data on SAFA and beta-carotene levels suggest that among NW, ST may have lower carbohydrate, vegetable and fruit intakes than those of NT. Although a reduced intake of vegetables has been reported for ST^45, 46^, no univocal data have been found for macronutrient intake based on PROP sensitivity. Some studies showed a preferential consumption of fats for NT^6^, but others did not confirm these data^16^; the contradiction suggests that these controversial findings may be due to different variables, such as ethnicity and traditional dietary behaviour, and leaves the issue unresolved. However, these differences were not evident in OB among PROP groups. However, although the decrease in beta-carotene is likely due to a decrease in vegetable and fruit intake, the increase in DNL biomarkers may be related to not only an excess carbohydrate intake but also other confounding factors related to metabolic disorders associated with obesity^47^. In addition, we did not find significant changes in the biomarker of dietary n-3 highly polyunsaturated fatty acid (HPUFA), the omega-3 index^36^, nor those for dairy products (the pentadecanoic acid^34^), indicating that at least at the qualitative level, ST did not differ from NT in terms of these nutritional habits as previously suggested^21^.

However, the omega-3 index values clearly indicate that the population included in the study was deficient in n-3 HPUFAs. The optimal value for this parameter was suggested to be approximately 8^48^. Further investigation should be conducted to determine whether this deficiency may influence the observed difference in endocannabinoid levels. In fact, studies of experimental animals^49–51^ and humans^52, 53^ showed that n-3 HPUFA intake modulates endocannabinoid levels. In addition, dairy products, particularly cheese enriched in conjugated linoleic, vaccenic and alpha-linolenic acids, have been shown to reduce AEA plasma levels^35^. However, in the present study, no significant changes were found in the plasma concentration of these fatty acids and pentadecanoic acid, suggesting a similar intake in NW and OB irrespective of PROP sensitivity. In addition, because dairy products are one of the major source of SAFA in the Italian population^44^, these data further confirm that the increase in SAFA in the lipid erythrocytes of OB is not likely due to their increased dietary intake but instead may reflect increased DNL.

Therefore, these parameters do not explain the higher BMI associated with lower endocannabinoids found in ST than in NT in OB. In addition, the score for disinhibition does not seem to explain these differences associated with PROP taste sensitivity. Evaluating the impact of the dietary pattern on lipid metabolism and body composition is extremely challenging due to the intrinsic difficulty of assessing food intake, especially in obese subjects, irrespective of the methodology used^54^, and the assessment of different fatty acid intake is even more complicated because of the high variability of fatty acid profiles, particularly in food of animal origin where the animals’ diet greatly influences tissue fatty acid content. Therefore, the use of circulating nutritional biomarkers may result in a more valid approach for objective estimates of dietary exposure than subjective self-reported food records.

MT represent the majority of the Caucasian population with a frequency of approximately 50%; in contrast, ST and NT account for approximately 25% each^1^. MT are probably the most protected from changes in metabolic parameters and have a more balanced dietary pattern and endocannabinoid profile that embodies the most physiologically advantaged population. However, a large part of the population (ST and NT) may have different metabolic and body composition consequences because of divergent and unbalanced dietary patterns. This possibility highlights the importance of identifying the PROP status when formulating individual dietary recommendations to maintain a balanced diet and thereby a proper body composition.

In conclusion, our data show that PROP taste sensitivity is associated (with an opposite trend in OB than in NW) with different BMIs, circulating endocannabinoids and lipid parameters, which could be interrelated to changes in dietary patterns and body fat distribution. However, as shown in Table 4, the effect size and power calculated for each dependent variable after conducting two-way ANOVA, indicate that for the underpowered comparisons, the lack of ability to detect significance may be due to small sample sizes. In fact, the study led to the non-equal number of participants in each of the 6 study groups because at recruitments we considered the normal frequency of 25% ST 50% MT and 25% ST typical of Caucasian populations^1^. However, a similar number of participants was considered a convenient sample in several studies on this topic^9, 55–57^.

Nevertheless, this finding suggests that PROP taste sensitivity, by differently affecting food preference in NW and OB, determines long-term metabolic changes and thereby body composition. Therefore, the use of different nutritional and metabolic biomarkers along with the determination of PROP taste sensitivity may provide important tools to design efficient personalized nutritional strategies aimed at maintaining a healthy physiological body composition.

## Methods

### Participants

One hundred and ten Caucasian volunteers (34M and 76F) were recruited in the area of Cagliari, Italy. They were divided into two groups based on their BMI, resulting in random classification by age and gender: NW (BMI from 18–25 kg/m^2^) (*N* = 60; 19M, 41F) and OB (BMI from 30 to 50 kg/m^2^) (*N* = 50; 15M, 35F).

The exclusion criteria included major diseases (e.g., diabetes and kidney disease), pregnancy or lactation, food allergies, and the use of medications that interfere with taste or smell (e.g., steroids, antihistamines, and certain antidepressants). All participants were verbally informed regarding the procedure and the aim of the study. They reviewed and signed an informed consent form. The present study was conducted according to the guidelines of the Declaration of Helsinki of 1975 (revised in 1983), and all procedures involving human participants were approved by the ethical committee of the Brotzu Institution. The study has been registered at ClinicalTrials.gov with Identifier: NCT02729584. Written informed consent was obtained from all participants.

### Procedure

All participants were requested to abstain from eating, drinking and using oral care products or chewing gums for at least 8 h prior to the sample withdrawal and the screening test for PROP taste sensitivity. All participants were required to be in the test room 15 min before the beginning of the trials, and their weight (kg) and height (m) were recorded to calculate the body mass index (BMI) (kg/m^2^). A 4 ml sample of blood was collected (at 8.00 AM) from each participant. Samples were immediately centrifuged and stored at −80 °C until the analyses were completed as described below. Participants completed the TFEQ^33^ for the assessment of general eating attitudes. The questionnaire estimates three aspects of cognitive control of eating behaviour: dietary restraint, disinhibition and perceived hunger.

The participants were screened and classified according to PROP taster status through the impregnated paper screening test, which was previously tested for validity and reliability^58–60^, and strongly correlate with the taste receptor cell depolarization^61^. The intensity ratings for PROP or NaCl were collected using the Labelled Magnitude Scale (LMS), a 100-mm scale anchored with the phrases “barely detectable” to “strongest imaginable”^62^. After tasting each solution, the participants placed a mark on the scale corresponding to his/her perception of the stimulus. Participants were categorized as non-tasters if they rated the PROP disk lower than 13 mm on the LMS; they were categorized as supertasters if they rated the PROP disk higher than 67 mm on the LMS. All other participants were classified as medium tasters^58^. Based on their taster group assignments, 38.33% of the NW were NT (*N* = 23; 8M, 15F), 46.67% were MT (*N* = 28; 10M, 18F), and 15% were ST (*N* = 9; 1M, 8F), whereas 26% of the OB were NT (*N* = 13; 7M, 6F), 50% were MT (*N* = 25; 7M, 18F), and 24% were ST (*N* = 12; 2M, 10F).

### Lipid analyses

The acetonitrile (CH_3_CN), methanol (CH_3_OH), chloroform (CHCl_3_), n-hexane (C_6_H_14_), ethanol (C_2_H_5_OH), and acetic acid (CH_3_COOH) were HPLC grade and purchased from Sigma Chemicals Co. (St. Louis, MO, USA). All standards of fatty acid standards were purchased from the same company.

Ascorbic acid, potassium hydroxide (KOH), and hydrochloric acid (HCl) were purchased from Carlo Erba, Milano, Italy. Deferoxamine mesylate was purchased from CIBA-Geigy (Basel, Switzerland). Internal deuterated standards for the AEA, 2-AG, palmitoylethanolamide (PEA) and oleoylethanolamide (OEA) quantification by isotope dilution ([2H]8AEA, [2H]52AG, [2H]4 PEA, and [2H]4 OEA) were purchased from Cayman Chemicals (MI, USA).

The total lipids were extracted by the method of Folch^63^. Briefly, samples of human plasma (1 ml) or erythrocytes were each homogenized into a 2:1 chloroform-methanol solution containing 2 μg of vitamin E and deuterated AEA (200 ng), 2-AG (300 ng), OEA (200 ng), and PEA (100 ng).

An aliquot of the plasma lipid extract was used for HPLC separation to determine the total free fatty acids as described previously^64^. The total lipid quantification was performed by the method of Chiang^65^.

### Fatty acid analysis

An aliquot of the erythrocyte lipid fraction of each sample was mildly saponified, and the fatty acids were analysed by HPLC (an Agilent 1100 HPLC system with a diode array detector, Palo Alto, Calif., USA) as previously described^66^. Spectra (195–315 nm) of the eluate were obtained every 1.28 s and were electronically stored. These spectra were acquired to confirm the identified HPLC peaks^67^.

Because SAFA are transparent to UV, after derivatization, they were measured as fatty acid methyl esters using a gas chromatograph (Agilent, Model 6890, Palo Alto, CA) as described previously^49^.

### Beta-carotene analysis

The results for other lipid parameters prompted us to analyse beta-carotene as a biomarker of vegetable and fruit intake. Therefore, due to the limited availability of the remaining samples, we were able to perform the analysis in only a subgroup of NW (*N* = 47) and OB (*N* = 38). An aliquot of the lipid fraction was analysed by HPLC with an array detector as previously described^68^.

### Analysis of the endocannabinoids and their congeners

Aliquots of the organic phase (chloroform) containing extracted lipids of human plasma samples were evaporated to dryness under a vacuum and reconstituted with 0.3 ml of 100% methanol.

Quantification of AEA, 2-AG, PEA and OEA was conducted by liquid chromatography-atmospheric pressure chemical ionization-mass spectrometry (LC-APCI-MS) and using selected ion monitoring (SIM) at M + 1 values for the four compounds and their deuterated homologues as previously described^69^.

### Statistical analyses

The data are expressed as the mean ± SD or SEM as specified in the legends. The differences between two groups were assessed using an unpaired, two-tailed Student’s t-test. The data sets involving more than two groups were assessed by ANOVA, and specifically, a two-way ANOVA was used to evaluate the differences for all parameters related to the PROP status in NW and OB. To evaluate whether the possible differences were due to a different gender distribution, we ran the same data analysis while considering only women. A two-way ANCOVA confirmed the association between PROP status and endocannabinoids after controlling for BMI and waist/hip ratio. Post hoc comparisons were conducted with Newman-Keuls test. Data with different superscripted letters were significantly different according to the post hoc ANOVA analysis. Chi-squared was used to evaluate whether the frequency of participants with T2D, was different among PROP taster groups. Post hoc statistical power analysis was conducted by using SPSS software (SPSS Inc. Headquarters, Chicago, IL, USA) to evaluate the effect size and power for each dependent variable after conducting two-way ANOVA where PROP taste and NW /OB status were independent variables. The eta squared, as parameter showing the effect size, and power are reported for each dependent variable in Table 4.

The statistical analyses were conducted using STATISTICA for WINDOWS (version 6.0; StatSoft Inc., Tulsa, OK, USA). *P* values < 0.05 were considered significant.
